# Minimal Manifestation Status Indicates a Stable State in Myasthenia Gravis: A Quantitative Study

**DOI:** 10.3389/fneur.2022.880045

**Published:** 2022-05-23

**Authors:** Ping Jiang, Jie Li, Hong-Yan Li, Bin Zhang, Yao-Xian Yue, Su-Yun Wang, Xi-Cun Zi, Shuang-Shuang Liu, Yi-Fan Li, Li-Dong Jiao, Hai-Feng Li

**Affiliations:** ^1^Department of Neurology, Xuanwu Hospital, Capital Medical University, Beijing, China; ^2^Department of Neurology, The Sixth Affiliated Hospital of Sun Yat-sen University, Guangzhou, China; ^3^Department of Neurology, Qilu Hospital (Qingdao), Cheeloo College of Medicine, Shandong University, Qingdao, China

**Keywords:** myasthenia gravis, minimal manifestation status, quantitative myasthenia gravis score, myasthenia gravis activities of daily living, quality of life for myasthenia gravis

## Abstract

**Introduction:**

Minimal manifestation (MM) or better was recommended as the treatment goal for myasthenia gravis (MG). The sustainability of this status has not been described quantitatively in patients who had attained or are close to it.

**Methods:**

Patients who were with no or slight impact on daily living were recruited and followed at baseline and 3, 6, and 12 months. The included patients were classified into 3 post-intervention status (PIS) categories: remission (R), MM, and slight impact (SI). The proportion of patients belonging to real-time (not considering the intervals between assessments) and sustained (considering the intervals between assessments) PIS categories was compared at each follow-up. A sensitivity analysis (SA) cohort was established by including patients with PIS categories in all four follow-ups. The QMGS, MG-ADL, and MG-QOL15 scores in patients belonging to each PIS category at each follow-up were compared. The sustainability of the R/MM status was examined and correlated with real-time R/MM status at follow-ups.

**Results:**

At baseline, 376 patients could be classified, including 55 as R (14.2%), 209 as MM (54.0%), and 112 as SI (28.9%). In the whole cohort, 68.8–89.7%, 71–76.7% and 19.8–77.1% of the patients classified into real-time R, MM, and SI categories remained unchanged in each follow-up compared with the previous follow-up. The proportion of patients belonging to each real-time or sustained R/MM status at the three follow-ups was 89.7–92.1 or 60.8–67. In the SA cohort, at least 86.4% of the baseline R/MM patients remained in R/MM status till 12 months. There were no differences in keeping real-time R/MM status at 6 or 12 months between patients with and without sustained R/MM status at 3 and 6 months. There were differences in the QMGS, MG-ADL, and MG-QOL15 scores among patients belonging to each real-time category at baseline and follow-ups, ranking as R < MM < SI. The same trend was observed in patients belonging to each sustained PIS category with smaller scores than the same items of real-time categories.

**Conclusion:**

The sustainability of the R/MM status was confirmed. The R/MM status indicated a stable state of MG. The QMGS, MG-ADL, and MG-QOL15 scores may provide a quantitative reference for these PIS.

## Introduction

In the international consensus guidance for managing myasthenia gravis (MG), minimal manifestation (MM) status or better was recommended as the main component of the treatment goal (1). MM refers to no symptoms or functional limitations from MG but some weakness on examination of some muscles (2). Remission and MM are the mildest end in the post-intervention status (PIS) classification. However, the proportion of patients achieving complete stable remission was only 7–20% and has not improved greatly compared with the 1940s (3). In 2011, Utsugisawa et al. proposed a practical goal of achieving “MM or better” status as the treatment goal (4). The sustainability of this status has not been described quantitatively during the follow-up in patients who had attained or been close to this status yet.

The current definition of MM status relies exclusively on patients' assessments of their symptoms and the impact on their daily living. There are few studies to provide a qualitative reference for the definition of the MM status. The quantitative MG score (QMGS), the MG activities of daily living (MG-ADL), and the 15-item MG quality of life scale (MG-QOL15) are validated measures in the evaluation of MG. One study reported the relevant QMGS and MG-QOL15 in remission or MM status (5). Other similar definitions have been defined recently. In “Patient-acceptable symptom states”, the ranges and thresholds of QMGS, MG-ADL, and MG-QOL15 were reported (6). “Minimal symptom expression” was defined as MG-ADL total score of 0–1 or MG-QOL15 total score of 0–3 as the thresholds (7).

In this study, we recruited MG patients who reported no or slight impact on their daily living at baseline, and described the changes and sustainability of the PIS from the baseline through 3, 6, and 12 months after inclusion, and explored the ranges and thresholds of the QMGS, MG-ADL, and MG-QOL15 scores in patients belonging to each PIS classification.

## Patients and Methods

### Included Patients

Patients were recruited consecutively and followed up from March 2017 to May 2019 at the Qilu Hospital of Shandong University (Qingdao). The diagnosis of MG was based on: (1) typical symptoms of fluctuating muscle weakness; (2) positive result of fatigue test; (3) unequivocal positive result of neostigmine test; (4) positive AChR antibody or positive MuSK antibody or amplitude decrement >10% on low-frequency RNS. The included patients should have 1, 2, and 3 as the essential conditions for the diagnosis, and at least one item in 4 as the supporting conditions. The patients were on symptomatic treatment and/or immunosuppressive treatment or were not on any treatments for MG in the setting of the outpatient management. The patients were requested to report their symptoms and the impact of symptoms on their daily living and were included in this study when they were asymptomatic, symptomatic with no impact or slight impact (SI) at the baseline. The patients who reported baseline moderate/severe impact (MSI) on daily living were excluded from this study. The patients with severe anxiety, depression, cognitive impairment, and poor understanding or cooperation in the assessment of QMG, ADL, and QOL were also excluded.

Data on clinical features and treatments of the included patients were collected, including gender, age of onset, current age, disease duration and current clinical classification (ocular or generalized), pyridostigmine bromide (PB) dose, corticosteroid (CS) dose (as prednisone equivalent dose), type and dosage of immunosuppressants (IS), and at each follow-up were recorded. This study was approved by the ethics committee of the hospital, and informed consent has been obtained from all MG patients.

### Follow-Up and Data Acquisition

Patients were requested to be followed up at 90 ± 20-day intervals, spontaneous follow-ups were encouraged as needed when their condition changed. At each scheduled follow-up, patients were required to report which impact category they belong to (1) asymptomatic; (2) symptomatic with no impact on daily living, (3) symptomatic with SI on daily living; (4) symptomatic with MSI on daily living. Then, an assessment of MG-ADL and MG-QOL15 was conducted with the assistance of an experienced physician or nurse. Necessary explanations were allowed only when the patients inquired about some confusion in the understanding of the scales. In case of confusion on whether some conditions were caused by MG, the assessment persons helped them to analyze based on their reported symptoms, medical history, and relevant physiological examinations (e.g., dyspnea due to asthma), but avoided replacing their judgment. Meanwhile, the judgment of the accompanying family members should be strictly prohibited to avoid affecting the judgment of patients themselves. The self-report of impact categories, MG-ADL and MG-QOL15 was based on the status of patients most of the time for the last 10 days before each follow-up, with regular PB taking as needed. As we found that almost 2/5 of the patients reported different impact categories when taking PB or not during the same period, the data at the follow-ups with irregular PB taking were not included in the analysis of MG-ADL and MG-QOL15. Subsequently, QMGS was assessed by the principal investigator (Li HF) when the last dose of PB was taken > 6 h before. The patients were asked whether they were too tired or hungry, and were encouraged to cooperate as much as possible during the QMGS assessment to avoid being judged as more severe than their daily condition. The data on the follow-ups with such conditions or poor cooperation due to medical or physiological interference (heart or lung diseases, electrolyte disturbance, cervical spondylosis or lumbar spine lesions, pain) were not included in the analysis of QMGS.

### Modified PIS Classification

**Figure 1:**
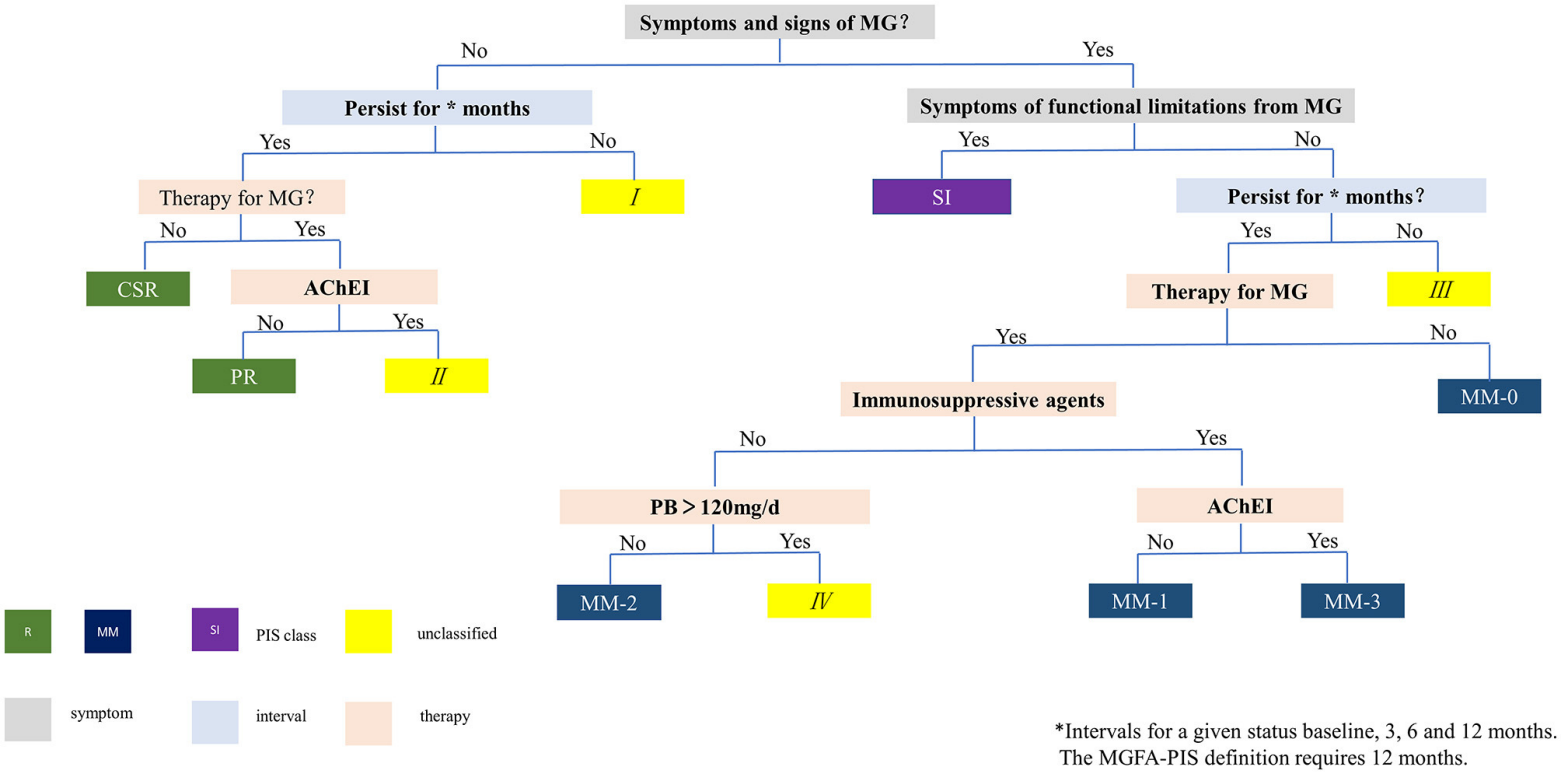
Flowchart for PIS classification. I, III: unable to maintain requested intervals (3, 6 or 12 months), therefore cannot be classified. II: no symptoms, normal fatigue test, but still on oral PB, therefore does not meet the definition of R. IV: PB dose >120 mg/d, therefore does not meet the definition of MM-2. MG, myasthenia gravis; PIS, post-intervention status; AChEI, acetylcholinesterase inhibitor; PB, pyridostigmine bromide; R, remission; CSR, complete stable remission; PR, pharmacological remission; MM, minimal manifestation; SI, slight impact on daily living.

We made a flowchart of the modified PIS classification based on the principle of Mutually Exclusive Collectively Exhaustive (MECE) by combining the impact categories and relevant intervals between assessment, fatigue test, PB dosage, and immunotherapies (Figure 1). The included patients were classified according to this flowchart into 3 categories: (1) remission (R): no symptoms, no impact on daily living, normal fatigue test (slightly incomplete eye closure is allowed), with or without immunotherapies, but without PB. (2) minimal manifestation (MM): no or mild symptoms, no impact on daily living for most of the time, abnormal fatigue test (some patients reported mild symptoms but with normal fatigue test at assessment were included in this category), with or without immunotherapies, with or without PB. MM is further divided into MM-0, MM-1, MM-2, and MM-3 according to the definition of PIS (2). (3) Slight impact (SI): same as MM except for symptoms and slight impact on daily living for most of the time. Since all included patients were mild at baseline by their self-reported impact on daily living, and there was no consensus on the quantitative definition of U and I, this category included some patients who would be classified as U and I categories. The original PIS definition of MGFA requires a minimum of 1 year for the definition of the R and MM status. In this study, various definitions of PIS were set without considering the intervals between assessment and with considering intervals of 3, 6, and 12 months, to reflect the real-time and sustained PIS through the scheduled follow-ups.

### Statistical Analysis

Real-time and sustained R, MM, and SI status were recorded in patients with qualified data at baseline and the three follow-ups. Patient characteristics, treatment, and QMGS, MG-ADL, and MG-QOL15 of the patients belonging to the three categories on each follow-up were described. For quantitative data, the Kruskal–Wallis test was used for comparison among multiple groups, and the Mann–Whitney U test was used for comparison between two groups. For categorical data, the chi-square test or Fisher's exact test was used for comparison. A sensitivity cohort was established by including patients with all eligible follow-ups and relevant data of PIS categories. Optimal cutoffs between R/MM and SI were generated with the receiver operating characteristic (ROC) curve. A two-tailed *p* < 0.05 was considered statistically significant. SPSS 23.0 software was used for statistical analysis.

## Results

### General Characteristics of Enrolled Patients

According to inclusion and exclusion criteria based on impacts on daily living, a total of 442 patients were included and followed. A total of fifty-five patients were excluded: 2 patients due to conflicting scores of ADL and QOL because of poor understanding of the scales, 1 patient due to pre-existing severe depression, 28 patients due to incomplete information (12 missing most of the key information, 3 with questionable QMGS records, 13 missing the impact categories at ≥ half of their scheduled follow-ups), 4 patients due to ambiguous impact categories, 18 patients cases due to not meeting the target impact categories at baseline when inspected retrospectively, and 2 patients whose MG diagnosis was excluded. A total of 387 patients were included in the final analysis. Among them, there were 169 males and 218 females, the current age on inclusion ranged from 15 to 87 years (48.7 ± 16.1 years), and the disease duration ranged from 7 days to 41 years (median 28.5 months, interquartile range 76 months). The onset age was 44.2 ± 18.4 years (41.8 ± 19.2 years for females and 48.8 ± 17.2 years for males).

At baseline, 376 patients could be classified into three categories, including 55 as R (14.2%), 209 as MM (54.0%), and 112 as SI (28.9%) (Table 1). The unclassified patients and the reasons are shown in Table 1. There was no difference in age, gender, and disease duration among patients belonging to the three categories. The proportion of patients with pure ocular involvement was higher in MM patients than in SI patients and was higher in MM-0–1 patients than in MM-2–3 patients. The proportion of treatment-naive (never being treated with immune therapies) patients was similar in R, MM, and SI patients (Table 2). There were no differences in baseline CS dosage between the MM patients and SI patients, but significantly higher than in R patients, and significantly higher in MM-2–3 patients than in MM-0–1 patients. There was no difference in the proportion of patients with IS usage among patients of the three categories, but significantly higher in MM-2–3 patients than in MM-0–1 patients. The proportion of patients with PB usage was lower in MM patients than in SI patients (Table 3).

**Table 1:** Patients belonging to each PIS category at baseline and each follow-up in the whole cohort and the sensitivity analysis cohort (number, %).

	**Baseline**	**3 months**		**6 months**		**12 months**	
		**Real-time**	**Sustained**	**Real-time**	**Sustained**	**Real-time**	**Sustained**

**W cohort**	***n** **=*** **387**	***n** **=*** **288**		***n** **=*** **226**		***n** **=*** **166**	
R	55 (14.2%)	88 (30.6%)	36 (12.5%)	86 (38.1%)	30 (13.3%)	62 (37.3%)	20 (12.1%)
**MM**	209 (54.0%)	170 (59.1%)	110 (38.2%)	120 (53.1%)	70 (31.0%)	91 (54.8%)	44 (26.5%)
MM-0	37 (9.6%)	6 (2.1%)	5 (1.7%)	0	0	0	0
MM-1	112 (28.9%)	134 (46.6%)	67 (23.3%)	111 (49.1%)	50 (22.1%)	81 (48.8%)	32 (19.3%)
MM-2	1 (0.3%)	0	0	0	0	0	0
MM-3	59 (15.2%)	30 (10.4%)	38 (13.2%)	9 (4.0%)	20 (8.8%)	10 (6.0%)	12 (7.2%)
SI	112 (28.9%)	19 (6.6%)	NA	16 (7.1%)	NA	11 (6.6%)	NA
MSI	0	5 (1.7%)	NA	2 (0.9%)	NA	2 (1.2%)	NA
I	NA	NA	56 (19.4%)	NA	57 (25.2%)	NA	42 (25.3%)
II	6 (1.6%)	5 (1.7%)	1 (0.3%)	1 (0.4%)	0	0	0
III	NA	NA	60 (20.8%)	NA	50 (22.1%)	NA	47 (28.3%)
IV	5 (1.3%)	1 (0.3%)	1 (0.3%)	1 (0.4%)	1 (0.4%)	0	0
R/MM	264 (68.2%)	258 (89.7%)	193 (67.0%)	206 (91.2%)	147 (65.0%)	153 (92.1%)	101 (60.8%)
Interval*	NA	NA	69 (24.0%)	NA	60 (26.5%)	NA	52 (31.3%)
**SA cohort**	***n** **=*** **151**	***n** **=*** **151**		***n** **=*** **151**		***n** **=*** **151**	
R	28 (18.5%)	51 (33.8%)	25 (16.5%)	58 (38.4%)	23 (15.2%)	58 (38.4%)	20 (13.2%)
**MM**	75 (49.7%)	89 (58.9%)	55 (36.4%)	82 (54.3%)	44 (29.1%)	81 (53.7%)	38 (25.2%)
MM-0	4 (2.6%)	0	0	0	0	0	0
MM-1	49 (32.5%)	76 (50.3%)	36 (23.8%)	76 (50.3%)	31 (20.5%)	72 (47.7%)	27 (17.9%)
MM-2	0	0	0	0	0	0	0
MM-3	22 (14.6%)	13 (8.6%)	19 (12.6%)	6 (4.0%)	13 (8.6%)	9 (6.0%)	11 (7.3%)
SI	47 (31.1%)	9 (6.0%)	NA	8 (5.3%)	NA	10 (6.6%)	NA
MSI	0	0	NA	2 (1.3%)	NA	2 (1.3%)	NA
I	NA	NA	27 (17.9%)	NA	36 (23.8%)	NA	38 (25.2%)
II	1 (0.7%)	2 (1.3%)	1 (0.7%)	1 (0.7%)	0	0	0
III	NA	NA	34 (22.5%)	NA	38 (25.2%)	NA	43 (28.5%)
IV	0	0	0	0	0	0	0
R+MM	103 (68.2%)	140 (92.7%)	102 (67.5%)	140 (92.7%)	96 (63.6%)	139 (92.1%)	89 (58.9%)
Interval*	NA	NA	39 (25.8%)	NA	45 (29.8%)	NA	50 (33.1%)

R, remission; MM, minimal manifestation; SI, slight impact on daily living; MSI, moderate/serious impact on daily living; NA, not applicable; W cohort, whole cohort; SA cohort, sensitivity analysis cohort. ^*^Interval: not the same as those of I and III, but as the intervals for sustained MM+R status.

**Table 2:** Generalized characteristics of patients belonging to each PIS category as the baseline.

	**R (*n =* 55)**	**MM (*n =* 209)**	**SI (*n =* 112)**	**MM 0-1 (*n =* 149)**	**MM 2-3 (*n =* 60)**
Current age	50.0 ± 19.9	48.8 ± 15.0	47.9 ± 15.6	48.0 ± 14.4	50.8 ± 16.6
Age at onset	45.7 ± 21.4	44.5 ± 17.6	43.0 ± 17.5	43.1 ± 17.3	47.0 ± 18.0
Female (%)	52.7% (29/55)	55.0% (115/209)	56.3% (63/112)	52.3% (78/149)	38.3% (23/60)
Duration (months)	26.0 (11.0–74.0)	24.0 (4.0–83.5)	37.5(14.0–109.8)	23.0 (4.5–97.0)	27.5 (3.0–60.8)
Current ocular type (%)	0	38.3% (80/209)	22.3% (25/112)^*a^	47.0% (70/149)	16.7% (10/60)**
Treatment naive (%)	3.6% (2/55)	12.0% (25/209)	14.3% (16/112)	16.8% (25/149)	0**
Duration of treatment naive (months)	22 &1	2.0 (1.0–9.0)	6.0 (0–26.0)^*b^	2.0 (1.0–9.0)	NA

PIS, post-intervention status; R, remission; MM, minimal manifestation; SI, slight impact on daily living. NA, not applicable. *Comparison among R, MM and SI groups (between MM and SI groups in the proportion of patients with ocular type) revealed significant difference, P < 0.05. ^a^Significant difference between MM and SI groups, P < 0.05. ^b^Significant difference between MM and SI groups, P < 0.05. **Significant difference between MM-0–1 and MM-2–3 groups, P < 0.05.

**Table 3:** The CS dosage and proportion of patients with IS or PB in patients belonging to each real-time PIS category at baseline and each follow-up.

	**R**	**MM**	**SI**	**MM-0–1**	**MM-2–3**
**CS dose (mg)**					
Baseline	15.0 (10.0–30.0)	40.0 (21.3–60.0)	50.0 (28.8–60.0)	35.0 (15.0–55.0)	55.0 (40.0–60.0)
(*n =* 299)	(*n =* 42)	(*n =* 168)	(*n =* 89)	(*n =* 111)	(*n =* 57)
3 months	25.0 (15.0–35.0)*	37.5 (25.0–46.3)*	38.2 ± 16.8	35.0 (25.0–45.0)	50.0 (37.5–60.0)
(*n =* 259)	(*n =* 78)	(*n =* 162)	(*n =* 19)	(*n =* 133)	(*n =* 29)
6 months	15.0 (10.0–20.0)*	25.0 (15.0–30.0)*	30.0 (17.5–50.0)	22.5 (15.0–30.0)*	31.1 ± 12.4*
(*n =* 212)	(*n =* 79)	(*n =* 118)	(*n =* 15)	(*n =* 109)	(*n =* 9)
12 months	10.0 (7.5–15.0)*	15.0 (10.0–30.0)*	20.0 (15.0–25.0)	15.0 (10.0–25.0)*	30.0 (20.0–47.5)
(*n =* 158)	(*n =* 58)	(*n =* 89)	(*n =* 11)	(*n =* 80)	(*n =* 9)
**IS %**					
Baseline	27.3% (15/55)	29.7% (62/209)	25.9% (29/112)	24.1% (36/149)	43.3% (26/60)
3 months	23.9% (21/88)	35.9% (61/170)	52.6% (10/19)*	31.4% (44/140)	56.7% (17/30)
6 months	23.3% (20/86)	48.3% (58/120)*	56.3% (9/16)	45.9% (51/111)*	77.8% (7/9)
12 months	25.8% (16/62)	52.7% (48/91)	36.4% (4/11)	48.1% (39/81)	90.0% (9/10)
**PB %**					
Baseline	0.0% (*n =* 55)	28.7% (60/209)	44.6% (50/112)	0.0% (*n =* 149)	100% (*n =* 60)
3 months	0.0% (*n =* 88)	17.6% (30/170)*	52.6% (10/19)	0.0% (*n =* 140)	100% (*n =* 30)
6 months	0.0% (*n =* 86)	7.5% (9/120)*	43.8% (7/16)	0.0% (*n =* 111)	100% (*n =* 9)
12 months	0.0% (*n =* 62)	11.0% (10/91)	36.4% (4/11)	0.0% (*n =* 81)	100% (*n =* 10)

CS, corticosteroid; IS, immunosuppressant; PB, pyridostigmine bromide; PIS, post-intervention status. *Significant difference in CS dose, IS % and PB % compared with the former follow-up in patients belonging to the same PIS category, P < 0.05.

### PIS Categories at Different Follow-Ups

In the whole cohort, the proportion of patients belonging to each real-time PIS category at baseline and 3, 6, and 12 months is shown in Table 1. 14.2–38.1% of the patients were classified as R, with a significant increase at 3 months compared with the baseline, and little change thereafter; 53.1–59.0% were classified as MM, with a slight increase at 3 months compared with the baseline, and little change thereafter; 6.6–28.9% were classified as SI, with a significant decrease at 3 months compared with the baseline, and little change thereafter. Only a small number of patients developed MSI (0.9–1.7%). 0.8–2.9% could not be classified, because the PB dosage did not meet the requirement for R (II) or MM-2 (IV) by the PIS definition, which was mainly observed at baseline (*n* = 11) and 3 months (*n* = 6).

The proportion of patients belonging to each sustained PIS category at 3, 6, and 12 months is shown in Table 1. 12.1–13.3% were classified as R, and 26.5–38.2% as MM, with no significant difference at the three follow-ups. 40.8–53.6% of the patients could not be classified, which slightly increased at 3 months compared with the baseline, with little change thereafter. The main reason is that the status cannot sustain to cover the requested intervals (I and III) (Table 1).

Since the whole cohort included the patients whose follow-up time was less than 12 months (loss of follow-up, or not yet reached 12 months due to the principal investigator moving from Qilu Hospital to Xuanwu Hospital) and patients whose impact category was missing at some follow-ups, we selected the patients who had all impact category information at the baseline and 3 follow-ups to establish a sensitivity analysis (SA) cohort. The proportion of patients belonging to each PIS category at baseline and each follow-up in this cohort was similar to those of the whole cohort. Only two patients were unable to be classified due to the PB dosage. The patients who could not be classified due to the requested intervals by PIS definition were similar in both cohorts (Table 1).

**Figure 2:**
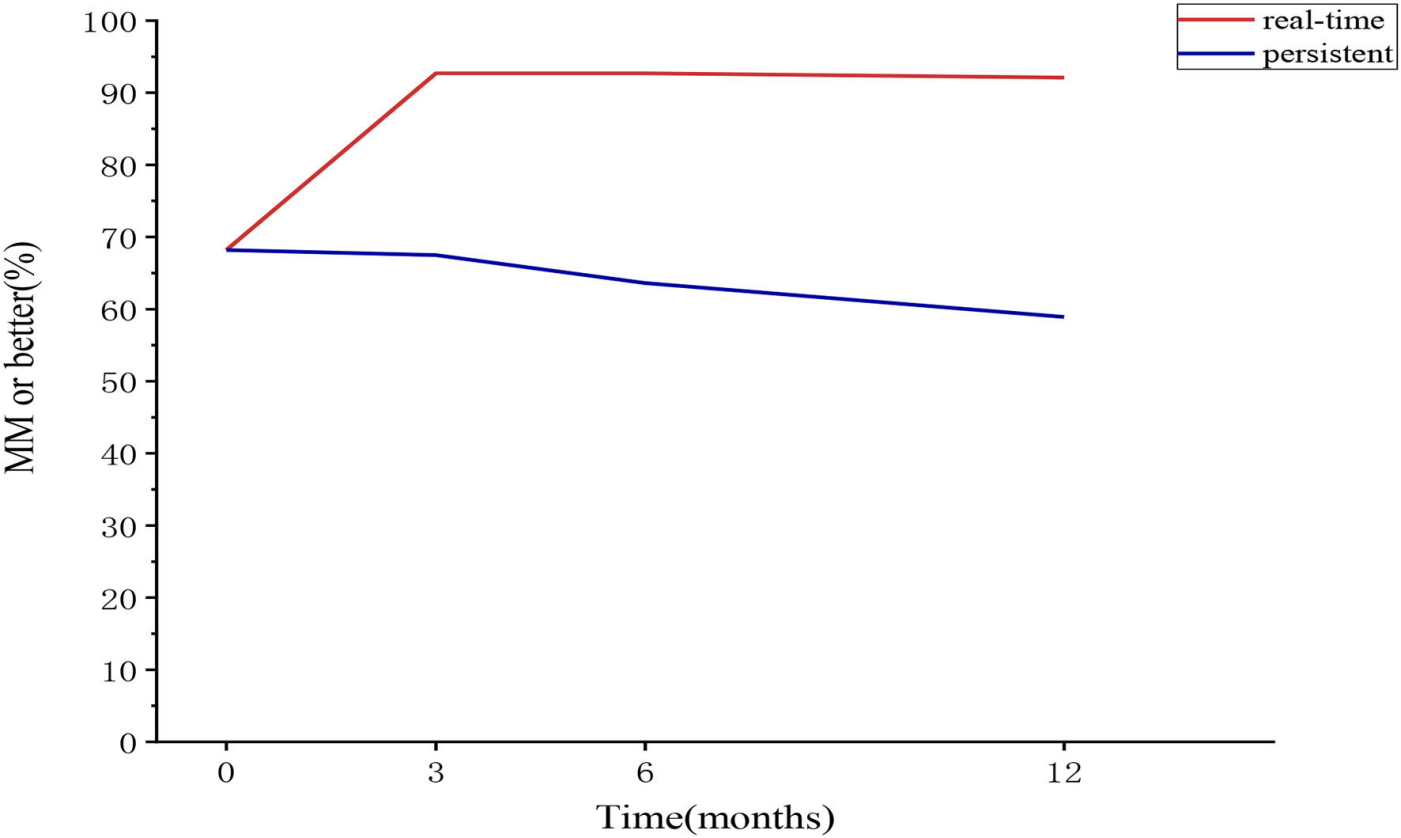
The proportion of patients belonging to the “MM or better” category at each follow-up in the sensitivity analysis cohort (real-time and sustained). MM, minimal manifestation.

The proportion of patients belonging to each real-time or sustained R/MM (MM or better) status at 3, 6, and 12 months was 89.7–92.1% and 60.8–67%, with no significant difference at the three follow-ups. About 0.8–2.9% and 25−31.3% of the patients could not be classified for each real-time or sustained R/MM status, with little change after 3 months (Table 1 and Figure 2).

### Changes of Treatment and Real-Time PIS Categories at Different Follow-Ups

The CS dosage in real-time R patients increased at 3 months, which is mainly related to the transfer of patients in the former MM and SI categories to this category. It fell to the baseline level at 6 months and further decreased at 12 months. There was decreasing trend in the CS dosage in the MM category at 3, 6 and 12 months compared with the former follow-ups. The CS dosage in the SI category showed an overall decreasing trend, although there was no difference in each follow-up compared with the previous one. The overall trend of CS dosage in the MM-0–1 sub-category was the same as that in the R category, although the CS dosage was larger than that in R category. The CS dosage in the MM-2–3 sub-category showing an overall decreasing trend, with little change after 6 months. There was no difference in the proportion of concurrent IS usage in the R category. The IS usage in the MM category at 6 and 12 months was higher than the baseline and 3 months. The IS usage in the SI category at 3 months was higher than the baseline and decreased after 6 months. The overall trend of IS usage in the MM-0–1 sub-category was the same as that of the MM category, and the overall trend of that in the MM-2–3 sub-category showed an increasing trend. The proportion of concurrent PB usage in the MM category was lower at 3 and 6 months than that of the previous follow-up, and there was no difference between 6 and 12 months. The overall trend of PB usage in the SI category decreased. There was no difference in PB usage at different follow-ups in the R, MM-0–1 and MM-2–3 categories (Table 3).

In the whole cohort, 68.8–89.7%, 71–76.7%, and 19.8–77.1% of the patients classified in real-time R, MM, and SI categories remained unchanged in each follow-up compared with the previous follow-up, respectively. The proportions of patients in MM category and SI category who remained unchanged at each follow-up were similar, except for the SI category which had a significant decrease at 3 months compared with the baseline. The proportions of patients in the sensitivity analysis cohort who remained unchanged at each follow-up were similar to those in the whole cohort (Table 4).

**Table 4:** Changes in patients belong to each real-time PIS category compared with the former follow-ups in the whole cohort and sensitivity analysis cohort.

**Baseline**	**R**			**MM**			**SI**		
**PIS**	**No change**	**Improvement**	**Worsening**	**No change**	**Improvement**	**Worsening**	**No change**	**Improvement**	**Worsening**
**Whole cohort**									
3 months	89.7% (35/39)	NA	10.3% (4/39)	71.0% (110/155)	26.4% (41/155)	2.6% (4/155)	19.8% (17/86)	76.7% (66/86)	3.5% (3/86)
6 months	78.9% (30/38)	5.3% (2/38)	15.8% (6/38)	76.7% (92/120)	13.3% (16/120)	10.0% (12/120)	76.9% (50/65)	20.0% (13/65)	3.1% (2/65)
12 months	68.8% (22/32)	15.6% (5/32)	15.6% (5/32)	72.9% (62/85)	14.1% (12/85)	12.9% (11/85)	77.1% (37/48)	6.3% (3/48)	16.6% (8/48)
**Sensitivity analysis cohort**									
3 months	89.3% (25/28)	NA	10.7% (3/28)	73.3% (55/75)	25.3% (19/75)	1.4% (1/75)	19.1% (9/47)	80.9% (38/47)	0
6 months	82.1% (23/28)	3.6% (1/28)	14.3% (4/28)	81.3% (61/75)	10.7% (8/75)	8.0% (6/75)	80.9% (38/47)	14.9% (7/47)	4.3% (2/47)
12 months	75.0% (21/28)	10.7% (3/28)	14.3% (4/28)	74.7% (56/75)	10.7% (8/75)	14.6% (11/75)	78.7% (37/47)	6.4% (3/47)	14.9% (7/47)

*PIS, post-intervention status; R, remission; MM, minimal manifestation; SI, slight impact on daily living; NA, not applicable*.

Since the proportions of patients who could be classified as R and MM and patients who remained unchanged in the R and MM categories were similar in the two cohorts (Tables 1, 4), the subsequent analysis of the QMGS, MG-ADL, and MG-QOL15 scores used the data from the whole cohort.

### Changes in Sustained PIS Categories at Different Follow-Ups

Using the data of the sensitivity analysis cohort, the sustainability of the PIS was explored (Table 5). About 87–92% of the patients belonging to the baseline R category remained in R category, with no difference through 3, 6, and 12 months; 73.3–86.4% of the patients belonging to the baseline MM category remained in the MM category, with the increasing trend through 3, 6, and 12 months; 92.7–99% of the patients belonging to the baseline R/MM category remained in the R/MM category, with no difference through 3, 6, and 12 months. Among the patients with changes in PIS categories, there were continuous improvements, improvement after initial worsening, or worsening after initial remission (Table 5). At least 86.4% (89/103) of the baseline R/MM patients remained in R/MM status at all follow-ups. The proportion of patients belonging to real-time and sustained R/MM status in the sensitivity analysis cohort is shown in Figure 2.

**Table 5:** Changes in patients belonging to each sustained PIS category compared with the former follow-ups in the sensitivity analysis cohort.

	**R**					**MM**				**R/MM**			
	**U**	**I**	**W**			**U**	**I**	**W**		**U**	**I**	**W**	
	**R**	**NA**	**MM**	**SI**	**MSI**	**MM**	**R/II**	**SI**	**MSI**	**R/MM**	**NA**	**SI**	**MSI**
3 mon	89.3% (25/28)	NA	10.7% (3/28)	0	0	73.3% (55/75)	25.3% (19/75)	1.4% (1/75)	0	99.0% (102/103)	NA	1.0% (1/103)	0
6 mon	92.0% (23/25)	NA	8.0% (2/25)	0	0	80.0% (44/55)	12.7% (7/55)	3.6% (2/55)	3.6% (2/55)	94.1% (96/102)	NA	3.9% (4/102)	2.0% (2/102)
12 mon	87.0% (20/23)	NA	13.0% (3/23)	0	0	86.4% (38/44)	6.8% (3/44)	6.8% (3/44)	0	92.7% (89/96)	NA	5.2% (5/96)	2.1% (2/96)

*PIS, post-intervention status; U, unchanged; I, improvement; W, worsening; NA, not applicable; R, remission; MM, minimal manifestation; SI, slight impact on daily living; MSI, moderate or serious impact on daily living*.

The real-time (6 or 12 months) R/MM status in the sensitivity analysis cohort of patients with sustained or non-sustained R/MM status of different intervals (3 or 6 months) is shown in Table 6. Whether there was sustained or non-sustained R/MM status in these patients, there was no difference in the proportion of attaining real-time R/MM status at 6 or 12 months.

**Table 6:** Sustained intervals of R/MM status and the real-time R/MM status at each follow-up in the sensitivity analysis cohort.

	**Sustained R/MM at 3 months** **(*n =* 102)**	**Non-sustained R/MM at 3 months** **(*n =* 1)**	**Sustained R/MM at 6 months** **(*n =* 96)**	**Non-sustained R/MM at 6 months** **(*n =* 7)**
R/MM at 6 months	96^a^	1		
Non-R/MM at 6 months	6	0		
R/MM at 12 months	97^b^	1	89^c^	7
Non-R/MM at 12 months	5	0	7	0

*R, remission; MM, minimal manifestation. ^a^p = 1.00; ^b^p = 1.00; ^c^p = 1.00*.

### QMGS, MG-ADL, and MG-QOL15 Scores in Patients Belonging to Each Category at Different Follow-Ups

**Figure 3:**
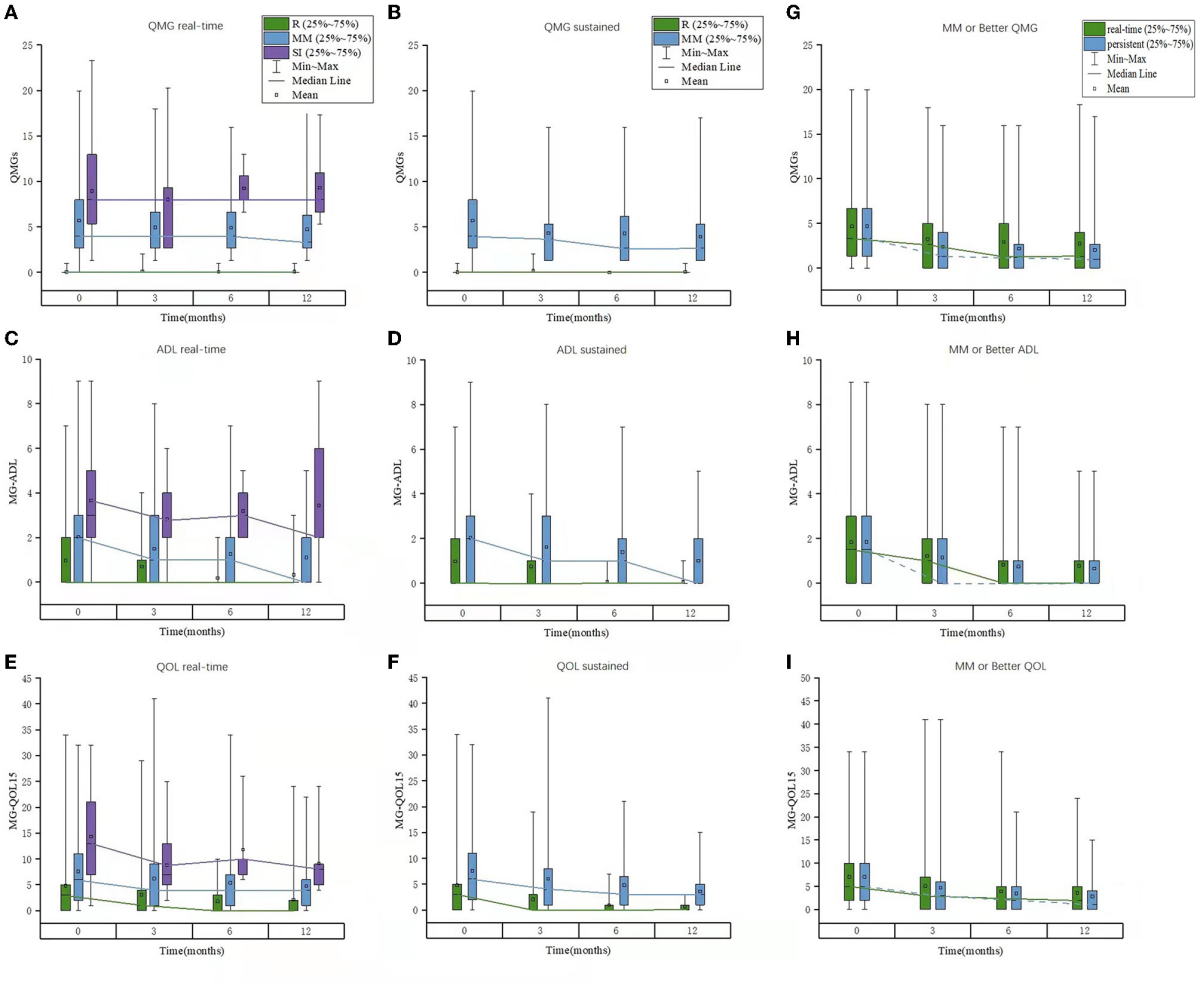
The scores of QMGS, MG-ADL, and MG-QOL15 in patients belonging to each PIS category or “MM or better” status at each follow-up in the whole cohort. **(A–C)** Real-time; **(D–F)** Sustained; **(E–I)** “MM or better” status. QMGS, quantitative myasthenia gravis score; ADL, activities of daily living; QOL, quality of life; PIS, post-intervention status; MM, minimal manifestation.

The patients with all data of QMGS, MG-ADL, and MG-QOL15 scores were used for quantification of each PIS category in the whole cohort. There were significant differences in the QMGS, MG-ADL, and MG-QOL15 scores (by mean ± SD or interquartile range) among patients belonging to each real-time category (R, MM, and SI), which were consistent at baseline and 3, 6, and 12 months of follow-ups. The three scores ranked from small to large as in the following categories R < MM < SI. The QMGS and MG-QOL15 scores were significantly lower in patients belonging to the MM-0–1 sub-category than those in the patients belonging to the MM-2–3 sub-category, whereas no differences were found in the MG-ADL scores between patients belonging to the two sub-categories. Although there was a significant decrease in some scores at 3 months compared with baseline, the overall trend of no significant changes was noted in patients belonging to the same category at each follow-up (Table 7 and Figure 3). The same trend was observed in patients belonging to each sustained PIS category during the follow-up, with smaller values than the same items of real-time categories and little difference between the MM-0–1 sub-category and MM-2–3 sub-category. A significant decrease in trend was noted in sustained MM-2–3 sub-categories than those of real-time MM-2–3 sub-categories.

**Table 7:** The scores of QMG, MG- ADL, and QOL in patients belonging to each PIS category at each follow-up in the whole cohort (real-time or sustained).

	**R**	***n***	**R/MM**	***n***	**MM**	***n***	**SI**	***n***	**MM-0–1**	***n***	**MM-2–3**	***n***
**Real-time**												
**Baseline**												
QMGs (*n =* 274)	0 (0–0.03)	35	3.33 (1.33–6.65)	192	3.99 (2.66–7.98)	1,57	7.98 (5.24–12.97)*	82	3.82 (2.66–5.49)	118	7.98 (3.99–11.97)**	39
ADL (*n =* 274)	0 (0–2.00)	35	1.50 (0–3.00)	192	2.00 (0–3.00)	157	3.66 ± 2.24*	82	2.00 (0.75–3.00)	118	2.00 (0–3.00)	39
QOL (*n =* 274)	3.00(0–5.00)	35	5.00 (2.00–10.00)	192	6.00 (2.00–11.00)	157	13.00 (7.00–21.00)*	82	6.00 (2.00–10.00)	118	9.00 (3.0–12.00)**	39
**3 months**												
QMGS (*n =* 182)	0 (0–0.03)	61	2.66 (0–4.99)†	171	3.99 (2.66–6.65)	110	7.98 (2.66–9.31)*	11	3.66 (2.66–6.65)	86	5.99 (2.99–9.31)**	24
ADL (*n =* 182)	0 (0–1.00)	61	1.00 (0–2.00)†	171	1.00 (0–3.00)†	110	2.82 ± 1.78*	11	1.00 (0–2.00)†	86	1.5 (0–3.00)	24
QOL (*n =* 182)	1.00 (0–4.00)	61	3.00 (0–7.00)†	171	4.00 (1.00–9.00)†	110	8.82 ± 6.60*†	11	3.50 (1.00–8.00)†	86	7.00 (3.00–13.00)**	24
**6 months**												
QMG (*n =* 139)	0 (0–0.02)	55	1.33 (0–5.07)	134	3.99 (2.66–6.65)	79	7.98 (7.32–11.81)*	5	3.99 (2.66–6.65)	75	8.48 (3.58–14.38)	4
ADL (*n =* 139)	0 (0)	55	0 (0–1.00)†	134	1.00 (0–2.00)	79	3.00 (2.00–4.50)*	5	1.00 (0–2.00)	75	0.50 (0–3.25)	4
QOL (*n =* 139)	0 (0–3.00)†	55	2.50 (0–5.00)	134	4.00 (1.00–7.00)	79	10.00 (6.50–18.00)*	5	4.0 (1.00–7.00)	75	3.50 (0.50–17.00)	4
**12 months**												
QMG (*n =* 139)	0 (0–0.02)	57	1.33 (0–3.99)	132	3.33 (2.66–6.32)	75	7.98 (6.65–10.98)*	7	2.66 (1.33–5.32)	66	5.32 (3.66–15.30)**	9
ADL (*n =* 139)	0 (0–0.50)	57	0 (0–1.00)	132	0 (0–2.00)	75	2.00 (2.00–6.00)*	7	0 (0–2.00)	66	2.00 (0–3.50)	9
QOL (*n =* 139)	0 (0–2.00)	57	2.00 (0–5.00)	132	4.00 (1.00–6.00)	75	8.00 (5.00–9.00) *	7	3.00 (1.00–6.00)	66	7.89 ± 3.98**	9
**Sustained**												
**3 months**												
QMGS (*n =* 125)	0 (0–0.03)	23^§^	1.33 (0–3.99)†	125	3.66 (1.33–5.32)†	67^§^	NA		2.66 (1.33–3.99)	39	4.83 (2.66–6.65)**†	28
ADL (*n =* 125)	0 (0–1.00)	23^§^	0 (0–2.00)†	125	1.00 (0–3.00)†	67^§^	NA		1.00 (0–3.00)†	39	1.00 (0–3.00)	28
QOL (*n =* 125)	0 (0–3.00)†	23^§^	3.00 (0–6.00)†	125	4.00 (1.00–8.00)†	67^§^	NA		3.00 (1.00–8.00)†	39	4.00 (1.25–10.75)†	28
**6 months**												
QMGS (*n =* 96)	0 (0–0.02)	21^§^	1.17 (0–3.66)	96	2.66 (1.33–6.24)	44^§^	NA		3.66 (2.33–6.65)	35	2.66 (1.33–4.49)†	9
ADL (*n =* 96)	0 (0)†	21^§^	0 (0–1.00)†	96	1.00 (0–2.00)	44^§^	NA		1.00 (0–2.00)	35	1.00 (0–1.50)	9
QOL (*n =* 96)	0 (0–1.50)	21^§^	2.00(0–5.00)	96	3.00 (1.00–6.75)	44^§^	NA		3.00 (0–6.00)	35	3.00 (2.00–9.50)	9
**12 months**												
QMGS (*n =* 87)	0 (0–0.02)	19^§^	1.00 (0–2.66)	87	2.66 (1.33–5.32)	35^§^	NA		2.66 (1.33–5.32)	25	2.66 (1.33–4.32)	10
ADL (*n =* 87)	0 (0)	19^§^	0 (0–1.00)	87	0 (0–2.00)	35^§^	NA		0 (0–2.00)	25	0.00 (0–1.00)	10
QOL (*n =* 87	0 (0–1.00)	19^§^	1.00 (0–4.00)	87	3.00 (1.00–5.00)	35^§^	NA		3.00 (1.00–5.00)	25	3.00 (1.00–5.75)	10

Mean ± standard deviation (SD) for data of normal distribution, median (interquartile range) for data of abnormal distribution. QMGS, quantitative myasthenia gravis score; ADL, activities of daily living; QOL, quality of life; PIS, post-intervention status; R, remission; MM, minimal manifestation; SI, slight impact on daily living; NA, not applicable. *Comparison among R category, MM category and SI category at the same follow-up, p < 0.05. **Comparison between MM-0–1 category and MM-2–3 category at the same follow-up, p < 0.05. †Comparison between each follow-up and the former follow-up in the same category, p < 0.05. ^§^The sum number of patients belonging to sustained R and MM categories were not equal to the number of patients belonging to sustained R/MM status due to R/MM contained the patients converted between the two categories.

The range of the scores was larger in some patients. For example, we used the real-time QMGS > 12 to show the proportion of patients with higher scores. At baseline, 12 out of 157 MM patients had higher QMGS (13–20), and their disease duration was 15–290 months. At 3 months, 5 out of 110 MM patients had higher QMGS (13–18), with a duration of 24–179 months. At 6 months, 3 out of 79 MM patients had higher QMGS (all 16), with a duration of 11–116 months. And at 12 months, 5 out of 75 MM patients had higher QMGS (13–18), with a duration of 74–197 months.

The cutoffs of QMGS, MG-ADL, and MG-QOL15 scores between the R/MM and SI status were generated with the ROC curve. The sensitivity was 76.8–100% and the specificity was mostly 61.4–88.5% (Table 8).

**Table 8:** Cutoffs of QMGS, MG-ADL, and MG-QOL15 between “MM or better” and SI at each follow-up in the whole cohort.

	**AUC (95%CI)**	***P*-Value**	**Optimal cut-offs**	**Sensitivity**	**Specificity**	**Accuracy**
**Real-time**						
**Baseline**						
QMGS (*n =* 274)	0.756 (0.698–0.815)	<0.01	4.83	76.8%	64.1%	67.9%
ADL (*n =* 274)	0.737 (0.673–0.801)	<0.01	1.50	85.4%	50.0%	60.6%
QOL (*n =* 274)	0.771 (0.714–0.828)	<0.01	5.50	90.2%	50.5%	62.4%
**3 months**						
QMGS (*n =* 182)	0.809 (0.705–0.914)	<0.01	2.33	100%	49.7%	52.7%
ADL (*n =* 182)	0.769 (0.636–0.901)	<0.01	1.50	81.8%	67.8%	68.7%
QOL (*n =* 182)	0.725 (0.610–0.839)	0.013	4.50	81.8%	61.4%	62.6%
**6 months**						
QMGS (*n =* 139)	0.924 (0.868–0.980)	<0.01	6.49	100%	81.3%	82.0%
ADL (*n =* 139)	0.904 (0.833–0.974)	<0.01	1.50	100%	77.6%	78.4%
QOL (*n =* 139)	0.893 (0.818–0.969)	<0.01	5.50	100%	76.1%	77.0%
**12 months**						
QMGS (*n =* 139)	0.914 (0.859–0.969)	<0.01	4.99	100%	80.3%	81.3%
ADL (*n =* 139)	0.821 (0.649–0.996)	<0.01	1.50	85.7%	79.5%	80.0%
QOL (*n =* 139)	0.824 (0.734–0.913)	<0.01	3.50	100%	64.4%	66.2%
**Sustained**						
**3 months**						
QMGS (*n =* 125)	0.824 (0.734–0.913)	<0.01	3.50	100%	64.4%	72.1%
ADL (*n =* 125)	0.777 (0.646–0.908)	<0.01	1.50	81.8%	69.6%	70.6%
QOL (*n =* 125)	0.752 (0.640–0.863)	<0.01	4.50	81.8%	66.4%	67.6%
**6 months**						
QMGS (*n =* 96)	0.954 (0.911–0.997)	<0.01	6.49	100%	88.5%	89.1%
ADL (*n =* 96)	0.910 (0.844–0.977)	<0.01	1.50	100%	81.3%	82.2%
QOL (*n =* 96)	0.896 (0.816–0.976)	<0.01	5.50	100%	76.0%	77.2%
**12 months**						
QMGS (*n =* 87)	0.944 (0.895–0.993)	<0.01	4.66	100%	85.1%	86.2%
ADL (*n =* 87)	0.836 (0.665–1.000)	<0.01	1.50	85.7%	82.8%	83.0%
QOL (*n =* 87	0.876 (0.797–0.955)	<0.01	3.50	100%	72.4%	74.5%

*QMG, quantitative myasthenia gravis score; ADL, activities of daily living; QOL, quality of life; MM, minimal manifestation; SI, slight impact on daily living*.

## Discussion

To our knowledge, this is the first study to systematically follow the changes in PIS and relevant QMGS, MG-ADL, and MG-QOL15 scores in patients who reported no or slight impacts of their symptoms on daily living.

For the sustainability of PIS in these patients whose current severity was at the milder end of the disease spectrum, this study is aimed to explore whether commonly used severity scores can provide a quantitative reference for these statuses.

The baseline PIS categories and proportion of treatment-naive were representative. The sustainability of PIS was examined and analyzed along with treatment changes. For the real-time PIS categories, there was an increase in the R and MM patients and a significant decrease in SI patients during the initial 3 months. At baseline, 2.9% of the patients could not be classified due to the usage of PB and PB dosage beyond the definition of relevant PIS categories. The CS dosage in R patients increased due to the conversion of former MM or SI patients into R patients accordingly. The CS dosage in MM and SI patients was on the decreasing trend. The proportion of patients on IS was stable in R patients and increased in MM and SI patients. The proportion of patients on PB decreased in MM and SI patients, especially in MM patients, however, the decrease was only seen in MM-0–1 patients. Larger CS dosage and more IS and PB were used in MM-2–3 and SI patients compared with MM-0–1 and R patients. These indicated that some patients with more severe PIS may convert into milder PIS along with decreased SC dosage and PB usage with the aid of adding IS and some patients will continue to improve despite the tapering of treatments. However, some patients may still need intense immunological treatment. In our practice, we encouraged patients to taper and withdraw PB after improvement as early as possible. In this cohort, with the extension of follow-up, the patients had a better understanding of reducing PB dosage after improvement. The PB dosage did not meet the PIS definition in only 2 patients at 6 months and none of the patients at 12 months.

For the sustained PIS categories, there was a similar proportion of R and MM, with a slightly decreasing trend. Half of the patients could not be classified, mainly due to the conversion of PIS, including continuous improvement, improvement after initial worsening, or worsening after initial remission. Due to the sustainability of R and MM being poor, MM or better R/MM was analyzed in both cohorts. The sustained R/MM status was found in at least 60.8% and 58.9% of the patients in the whole and SA cohorts. The sustainability of the R, MM, and R/MM status in the SA cohort was similar to that in the whole cohort, indicating that a close follow-up could not change the PIS categories.

To further explore the sustainability of PIS, we analyzed the unchanged patients belonging to baseline R and MM in the SA cohort. In unchanged patients at 3 months, the proportion of being unchanged in the next follow-up was high, indicating a stable state in these patients. At least 86.4% of the baseline R/MM patients remained in the R/MM status at 12 months. Whether they were in sustained or non-sustained R/MM status at 3 or 6 months, they were still keeping the real-time R/MM status at 6 or 12 months. This indicated that the MM or better status was an indicator of a stable state of MG.

There were significant differences in the QMGS, MG-ADL, and MG-QOL15 scores among patients belonging to each real-time category (R, MM, and SI), which were consistent at baseline and 3-, 6- and 12-month follow-ups. The same trend was observed in patients belonging to each sustained PIS category during the follow-up. Moreover, the QMGS, MG-ADL, and MG-QOL15 scores were found smaller in patients of sustained PIS categories than those in patients of the same real-time categories, especially in the MM-2–3 sub-category, indicating a more stable disease state of MG in patients with sustained PIS. The optimal cutoffs between the R/MM and SI categories were satisfactory at most of the follow-ups. These facts indicated that the three commonly used scoring systems were eligible to provide a quantitative reference for the R and MM status.

In the Japanese study of PIS, the total QMGS score was reported as 2.6 ± 1.5, 3.0 ± 2.0, and 4.6 ± 2.4 and the total MG-QOL15 score was reported as 8.8 ± 9.7, 9.4 ± 10.5, and 11.5 ± 10.5 for the complete stable R, pharmacological R, and MM status (5). In the Canadian study, the total scores of QMGS, MG-ADL, and MG-QOL15 were reported as 4.28 ± 2.78, 1.04 ± 1.21, and 6.28 ± 7.45 for the patient-acceptable symptom states (6). The minimal symptom expression was defined as MG-ADL total score of 0–1 or MG-QOL15 total score of 0–3 (7) In our study, the total QMGS score was reported as 0 (0-0.03) and 3.99 (2.66–7.98), the total MG-ADL MG-ADL scores was 0 (0–2.00) and 2.00 (0–3.00), and the total MG-QOL15 score was 6.00 (2.00–11.00) for the R and MM status. The difference between the four studies might be due to the difference in details in the definition of R and MM, and the subjective experience in the MG symptoms and the severity of the included patients.

This study has its strength in its representative distribution of baseline real-time PIS categories in a real-world cohort and the large sample size. Detailed changes in PIS categories and their relation to the changes in treatment were shown by comparison through the follow-up time points. However, several limitations should be emphasized. The number of SI patients was small at the last two follow-ups due to inadequate or loss of follow-up, which might overestimate the accuracy of cutoffs between the R/MM and SI status. However, the sensitivity and specificity were intrinsic determinants of the accuracy, which was relatively satisfactory. Several patients with high QMGS, MG-ADL, and MG-QOL15 were included, which might enlarge their ranges and IQRs. This reflected the real-world conditions of the patients, whose disease duration was found to be long, which might render them to tolerate their symptoms well subjectively. Moreover, although the agreement between QMGS and MG-ADL was found good in moderate or severe MG patients (8), the correlation between the two scores was weaker in patients who were in the MM status, demonstrating a “floor effect”. The disagreement was also found in mild patients in this study. We will further explore the factors associated with discordance between the physician-evaluated score (QMGS) and the self-reported MG-ADL or MG-QOL15. Furthermore, there might be effects from patients who dropped out from follow-up on the rates of PIS (particularly R status) at 12 months. However, because of the complex reasons for drop-out (due to neglect of minor fluctuation or random drop-out), a sensitivity analysis on the specific PIS categories and scores could not be conducted in light of the absence of drop-out reasons at the individual level.

In conclusion, the sustainability of R status was confirmed as poor. However, the sustainability of R/MM status was confirmed as excellent. The R/MM status indicated a stable state in MG patients. The QMGS, MG-ADL, and MG-QOL15 scores may provide a quantitative reference for these PIS.

## Data Availability Statement

The original contributions presented in the study are included in the article/supplementary material, further inquiries can be directed to the corresponding author/s.

## Ethics Statement

The studies involving human participants were reviewed and approved by Ethics Committee of Xuanwu Hospital, Capital Medical University; Ethics Committee of Qilu Hospital (Qingdao), Cheeloo College of Medicine, Shandong University. Written informed consent to participate in this study was provided by the participants' legal guardian/next of kin.

## Author Contributions

H-FL conceptualized and designed the study and revised the manuscript. PJ and JL interpreted the data and wrote the manuscript. H-YL, BZ, and Y-XY assisted in the follow-up and assessment of QMGS. S-YW and X-CZ assisted in the assessment of MG-ADL and MG-QOL15. S-SL, Y-FL, and L-DJ contributed to statistical analysis and manuscript revision. H-FL and Y-XY diagnosed, treated, recruited, and followed up with these patients in this study. All authors contributed to the article and approved the submitted version.

## Funding

This study was supported by the National Natural Science Foundation of China (Grant Nos. 81771362 and 82171397 to H-FL).

## Conflict of Interest

The authors declare that the research was conducted in the absence of any commercial or financial relationships that could be construed as a potential conflict of interest.

## Publisher's Note

All claims expressed in this article are solely those of the authors and do not necessarily represent those of their affiliated organizations, or those of the publisher, the editors and the reviewers. Any product that may be evaluated in this article, or claim that may be made by its manufacturer, is not guaranteed or endorsed by the publisher.
